# Cost avoidance analysis of outpatient parenteral antimicrobial therapy (OPAT) plan reconciliation for patients enrolled in value-based payment models at an academic hospital

**DOI:** 10.1017/ash.2026.10329

**Published:** 2026-04-01

**Authors:** Jennifer K. Ross, Kimberly D. Boeser, Matthew Hordyk, Bhupinder S. Manhani, Elizabeth B. Hirsch, Michael D. Evans, William D. Sieling, Susan E. Kline, Alison L. Galdys

**Affiliations:** 1 Department of Pharmacy, Fairview Pharmacy Services, M Health Fairview University of Minnesota Medical Center, Minneapolis, MN, USA; 2 Fairview Health Services, USA; 3 University of Minnesota College of Pharmacy: University of Minnesota Twin Cities, USA; 4 University of Minnesota Clinical and Translational Science Institute: University of Minnesota Twin Cities, USA; 5 University of Minnesota School of Medicine: University of Minnesota Twin Cities, USA

## Abstract

We previously characterized a reduction in unscheduled care following implementation of ID pharmacist review of outpatient parenteral antimicrobial therapy plans for patients discharging from acute care. In this report we demonstrate the cost avoidance that this intervention would be expected to generate for patients enrolled in value-based-care plans.

## Introduction

Effective delivery of outpatient parenteral antimicrobial therapy (OPAT) has many potential benefits, including reduced length of hospitalizations and hospital-associated conditions.^1^ OPAT is complex, however, and can exacerbate vulnerabilities in a health system’s ability to transition patients from acute to outpatient care. To mitigate the potential negative impact of OPAT on care transitions, our institution implemented Infection Diseases (ID) pharmacist review of all OPAT plans for patients who discharge from acute to ambulatory settings. This intervention, which included dose, frequency, and duration reconciliation and identification of the managing OPAT provider, was associated with reduced all-cause readmissions (pre: 39.1%; post: 33.1%; *P* < .01) and ED visits (pre: 22.3%; post: 17.9%; *P* = .03).^2^ The presence of a structured OPAT program has also been associated with reduced unscheduled care among OPAT recipients at other centers.^3,4^

Only a fraction of the clinical activities related to OPAT care are characterized as billable encounters based on the current Centers for Medicare and Medicaid Services (CMS) coding scheme.^5,6^ As a consequence, it is challenging to demonstrate the monetized value that multidisciplinary OPAT teams bring to a health system. Prior work to quantify the financial advantages of OPAT has estimated the acute care-related costs that are avoided by delivering antimicrobials in the outpatient setting.^7^ However, these analyses do not account for additional costs that are associated with unscheduled care after an index OPAT admission.

Value-based care (VBC) is a delivery model in which reimbursement is based on patient outcomes - and not based on the volume of services provided.^8^ Alternative payment models (APMs) are a type of VBC and depart from open-ended fee-for-service payments by imposing a degree of financial risk on the participating healthcare system.^8^ Within some APMs, such as downside risk contracts, there are financial disincentives for unscheduled care. In 2023, 28.5% of U.S. health care payments flowed through downside risk contracts.^9^ Adoption of APMs is anticipated to increase with the CMS Innovation Center aspiring to have all Medicare beneficiaries and most Medicaid beneficiaries in an APM plan by 2030.^9^ Highlighting the alignment between APM incentives and the outcomes of structured OPAT programs represents an opportunity to quantify the monetized value these programs bring to health systems.

We sought to analyze the potential financial impact of our historic OPAT cohort’s reduction in unscheduled care by comparing the charges among a cohort of patients whose OPAT plans were reviewed by an ID pharmacist upon discharge from acute care, an initiative that we launched on 6/15/2020, with a cohort of patients who were discharged before 6/15/2020 and whose OPAT plans were not reviewed.

## Methods

Our sample was derived from a prior retrospective cohort of 2,408 unique patients who received OPAT following discharge from the University of Minnesota Medical Center.^2^ For each patient, the hospitalization that was succeeded by OPAT was defined as the index OPAT admission.

We obtained charge data from our health system’s finance department. Financial data are only retained for 5 years, so for 1,067 patients in our cohort, financial data were not available. Our final cohort for the present analysis included 1,341 patients from 6/15/2019 to 6/14/2022: 577 from the preintervention period, and 764 from the postintervention period. We tabulated the charges that each patient incurred as a consequence of care in acute or emergency locations (ie charges related to ambulatory care were excluded) for 90 days before and including their index OPAT admission, and for 90 days after their index OPAT admission. We calculated the aggregate median and mean charges for the pre and postintervention cohorts, respectively. The differences in aggregated charges before and after an index OPAT admission were then calculated for the preintervention and postintervention cohorts, respectively, and the quantities of charges following an OPAT admission relative to quantities of charges leading up to and including an OPAT admission were compared.

## Results

**Figure 1.**
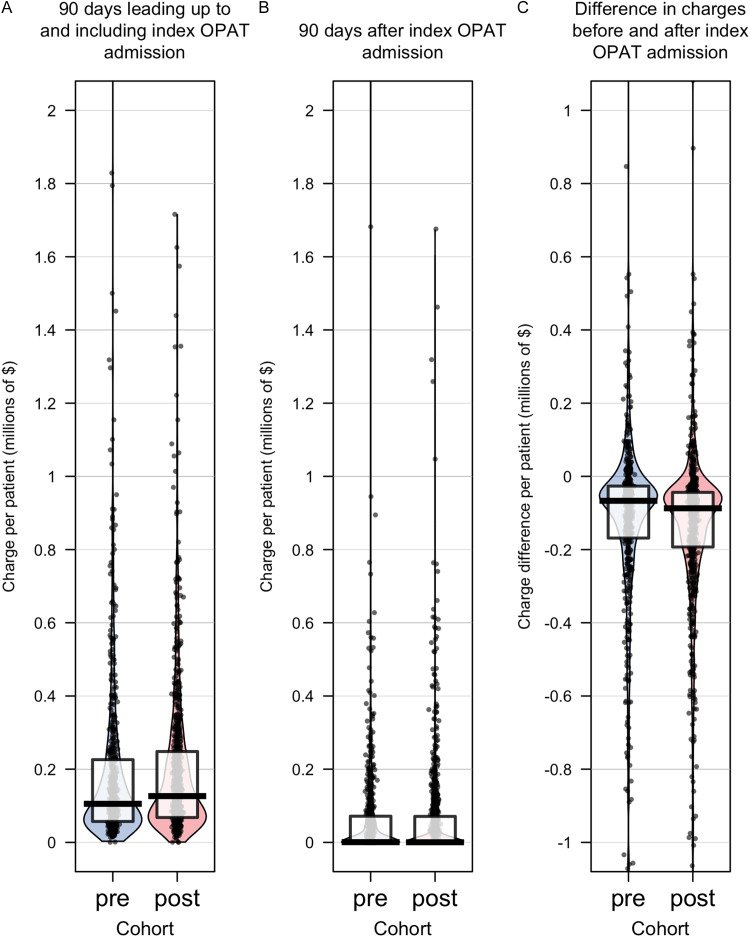
Violin and boxplots depicting charge data. During the 90 days before and including an index OPAT admission, the median charges per patient in the postintervention cohort were higher (Panel A). During the 90 days following an index OPAT admission, the median per-patient charges were not statistically significantly different for the preintervention cohort compared to the postintervention cohort, though the charges were numerically lower for the postintervention cohort (Panel B). The reduction in charges following an OPAT admission was greater in the postintervention cohort compared to the preintervention cohort (Panel C). Boxes span 25^th^–75^th^ percentiles and bars indicate medians.

The range of charges incurred before and after an index OPAT admission was widely variable in both the pre and postintervention cohorts. (Figure 1A, 1B). The aggregate charges for both the pre and postintervention cohort were higher for the 90 days leading up to and including an index OPAT admission compared to the 90 days following an index OPAT admission (Table 1). There was not a statistically significant difference in charges incurred by patients in the postintervention cohort during the 90 days following an index OPAT admission, though these charges were numerically lower (median $365 mean $66,906), than the preintervention cohort (median $666, mean $74,265) (Table 1). When examining the difference in median charges in the 90 days before and after an index OPAT admission, the postintervention cohort had a bigger difference—of $20,419 per patient—in incurred charges compared to the preintervention cohort (Table 1, Figure 1C).

**Table 1.** Central tendencies of per-patient charges for pre-intervention and post-intervention cohorts. OPAT, outpatient parenteral antimicrobial therapy; IQR, inter-quartile range, SD, standard deviation

	Pre-intervention			Post-intervention		Pre-intervention		Post-intervention		
	N	Median [IQR]	N	Median [IQR]	*P*-value	N	Mean (SD)	N	Mean (SD)	*P*-value
90 days leading up to and including index OPAT admission	577	105,361 [57,226, 226,070]	764	126,456 [68,053, 248,308]	.004	577	195,144 (253,009)	764	202,838 (220,405)	.56
90 days after index OPAT admission		666 [222, 71,623]		365 [235, 71,012]	.92		74,265 (210,130)		66,906 (159,670)	.48
Difference in 90-day charges before and after index OPAT admission		−66,739 [−168,514, −26,532]		−87,158 [−192,977, −43,720]	.001		−120,879 (303,102)		−135,932 (242,797)	.33

## Discussion

Delivering quality care for OPAT patients requires the time and expertise of a multidisciplinary clinician team. However, lack of financial support from institutions and via reimbursement is a frequent barrier to establishing and maintaining OPAT programs.^10^ In this investigation, we demonstrate numerically but not statistically significantly reduced charges related to unscheduled care after an index OPAT admission following the implementation of enhancements to our OPAT program. For patients enrolled in APMs, reduced unscheduled care charges are costs avoided by our institution’s affiliated health system. In our cohort for this analysis, approximately 17% were enrolled in an APM. If, in the future, 17% or more of our hospital’s average annual population of 950 OPAT patients also have APMs, it is possible that our intervention could yield an avoidance of between $48,611.50 and $1.18 million in annual financial penalties related to unscheduled care in OPAT recipients. At minimum, this cost avoidance would be expected to cover 0.25 full-time equivalent (FTE) ID pharmacist effort.

This study has several limitations. The charge data used in our analysis are proprietary and were provided without patient identifiers. As a consequence, we could not assess patient characteristics, nor could we identify which specific patients had health plans with downside risk contracts as an APM. The difference in post-OPAT admission charges between our cohorts did not reach statistical significance, so it is possible that future analyses would yield different results. However, even if the reduced charges we demonstrate did not actually translate to cost avoidance by our health system, we feel our results support OPAT plan review as a way to reduce overall healthcare costs. Charge data were inclusive only of acute and emergency care charges in our health system, so we could not quantify additional health system revenue that would be actualized by ambulatory charges for OPAT patients and that are not subject to penalty. Our conclusions regarding the cost avoidance that occurred because of reduced unscheduled care in our postintervention cohort does not account for shifting financial incentives within VBC models, including APMs. The onset of the COVID pandemic late in our preintervention period may have influenced care-seeking behavior and illness acuity among patients in our cohorts, and we could not account for this. Finally, our financial assessments are not standardized to a dollar valuation.

In summary, we present evidence that outcomes incentivized by VBC models align with outcomes facilitated by structured OPAT programs. Implementation of formal ID pharmacist reconciliation of OPAT plans is likely to generate significant cost avoidance among OPAT patients enrolled in health plans that utilize APMs.
